# On the nonlinear Hadamard-type integro-differential equation

**DOI:** 10.1186/s13663-021-00693-5

**Published:** 2021-03-15

**Authors:** Chenkuan Li

**Affiliations:** Department of Mathematics and Computer Science, Brandon University, Brandon, Manitoba R7A 6A9 Canada

**Keywords:** 34A08, 34A12, Hadamard-type fractional derivative, Hadamard-type fractional integral, Babenko’s approach, Multivariate Mittag-Leffler function, Absolute continuity, Banach’s contraction principle

## Abstract

This paper studies uniqueness of solutions for a nonlinear Hadamard-type integro-differential equation in the Banach space of absolutely continuous functions based on Babenko’s approach and Banach’s contraction principle. We also include two illustrative examples to demonstrate the use of main theorems.

## Introduction

The Hadamard fractional integration and differentiation are based on the *n*th integral of the form [1, 2]

$$\begin{aligned} \bigl(\mathcal{J}^{n}_{ a + , \mu } u\bigr) (x) = & x^{-\mu } \int _{a}^{x} \frac{d t_{1}}{t_{1}} \int _{a}^{t_{1}} \frac{d t_{2}}{t_{2}} \cdots \int _{a}^{t_{n - 1}} t_{n}^{\mu } u(t_{n}) \frac{d t_{n}}{t_{n}} \\ = & \frac{1}{(n - 1)!} \int _{a}^{x} \biggl(\frac{t}{x} \biggr)^{\mu } \biggl(\log \frac{x}{t} \biggr)^{n - 1} u(t) \frac{d t}{t} \end{aligned}$$

and the corresponding derivative

$$\begin{aligned}& \bigl(\mathcal{D}^{1}_{a + , \mu } u\bigr) (x) = \bigl(( \delta + \mu ) u\bigr) (x) = x u'(x) + \mu u(x),\quad \delta = x \frac{d}{d x}, \\& \mathcal{D}^{n}_{a + , \mu } u = \mathcal{D}^{1}_{a + , \mu } \bigl(\mathcal{D}^{n - 1}_{a + , \mu } u\bigr),\quad n = 2, 3, \ldots , \end{aligned}$$

where $\log ( \cdot ) = \log _{e} (\cdot )$, $0 < a < x < b$, and $\mu \in R$.

The fractional version of the Hadamard-type integral and derivative are given by

$$ \bigl(\mathcal{J}^{\alpha }_{ a + , \mu } u\bigr) (x) = \frac{1}{\Gamma (\alpha )} \int _{a}^{x} \biggl(\frac{t}{x} \biggr)^{\mu } \biggl(\log \frac{x}{t} \biggr)^{\alpha - 1} u(t) \frac{d t}{t},\quad \alpha > 0 $$

and

$$ \bigl(\mathcal{D}^{\alpha }_{a + , \mu } u\bigr) (x) = x^{-\mu } \delta ^{n} x^{\mu }\bigl( \mathcal{J}^{n - \alpha }_{ a + , \mu } u\bigr) (x), $$

where $n = [\alpha ] + 1$, and $[\alpha ]$ being integral part of *α*.

When $0 < \alpha < 1$, the fractional derivative turns out to be

$$\begin{aligned} \bigl(\mathcal{D}^{\alpha }_{ a + , \mu } u\bigr) (x) =& x^{-\mu } \delta x^{\mu }\bigl(\mathcal{J}^{1 - \alpha }_{ a + , \mu } u\bigr) (x) \\ =& \frac{1}{\Gamma (1 - \alpha )} x^{-\mu + 1} \frac{d}{d x} \int _{a}^{x} t^{\mu - 1} \biggl(\log \frac{x}{t} \biggr)^{- \alpha } u(t) \,dt. \end{aligned}$$

In particular, for $\alpha = 1$,

$$ (\mathcal{J}_{ a + , \mu } u) (x) = \bigl(\mathcal{J}^{1}_{ a + , \mu } u\bigr) (x) = \frac{1}{\Gamma (\alpha ) x^{\mu }} \int _{a}^{x} t^{ \mu - 1} u(t) \,dt, $$

which leads to defining the space ${X}_{\mu }[a, b]$ of those Lebesgue measurable functions *u* on $[a, b]$ for which $x^{\mu - 1} u(x)$ is absolutely integrable [2]:

$$ X_{\mu }[a, b] = \biggl\{ u : [a, b] \rightarrow C \mbox{ and } \lVert u \rVert _{X_{\mu }} = \int _{a}^{b} x^{\mu - 1} \bigl\vert u(x) \bigr\vert \,dx < \infty \biggr\} . $$

Let $\operatorname{AC}[a, b]$ be the set of absolutely continuous functions on $[a, b]$. Then it follows from [3] that

$$ u \in \operatorname{AC}[a, b] \quad \mbox{if and only if}\quad u(x) = u(a) + \int _{a}^{x} v(t) \,dt, \quad v(t) \in L[a, b]. $$

Obviously,

$$ \operatorname{AC}[a, b] \subset X_{\mu }[a, b]. $$

The latter is a Banach space under its norm. We further define the space

$$ \operatorname{AC}_{0}[a, b] = \biggl\{ u : u(x) \in \operatorname{AC}[a, b] \mbox{ with } u(a) = 0 \mbox{ and } \lVert u \rVert _{0} = \int _{a}^{b} \bigl\vert u'(x) \bigr\vert \,dx < \infty \biggr\} . $$

Clearly, $\lVert u \rVert _{0}$ is a norm in $\operatorname{AC}_{0}[a, b]$. Indeed, if $\lVert u \rVert _{0} = 0$ then $u(x) = u(a) = 0$. To show that $\operatorname{AC}_{0}[a, b]$ is complete, we assume $\{u_{n} (x) \}$ is a Cauchy sequence in $\operatorname{AC}_{0}[a, b]$, then we need to find a function $u(x)$ such that $u(x)$ is absolutely continuous and $u_{n} \rightarrow u$ under its norm. Since $\{u_{n} (x) \}$ is Cauchy in $\operatorname{AC}_{0}[a, b]$, we claim that $u_{n}(a) = 0$ and $\{u'_{n} (x) \}$ is Cauchy in $L[a, b]$. Hence, there exists $g \in L[a, b]$ such that $u'_{n} \rightarrow g $ in $L[a, b] $. Define

$$ u(x) = \int _{a}^{x} g(\tau ) \,d\tau . $$

Then $u(a) = 0$ and $u(x)$ is absolutely continuous on $[a, b]$, and

$$ \lVert u_{n} - u \rVert _{0} \leq \int _{a}^{b} \bigl\vert u_{n}'(x) - g(\tau ) \bigr\vert \,d\tau $$

converges to zero. Therefore, $\operatorname{AC}_{0}[a, b]$ is a Banach space.

### Lemma 1.1

*If* $\alpha > 0$, $\mu \in R$, *and* $0 < a < b < \infty $, *then the operator* $\mathcal{J}^{\alpha }_{ a + , \mu }$ *is bounded in* $\operatorname{AC}_{0}[a, b]$ *and*

$$ \bigl\lVert \mathcal{J}^{\alpha }_{ a + , \mu } u \bigr\rVert _{0} \leq \frac{C_{\mu }}{\Gamma (\alpha + 1)} \biggl[\log \biggl( \frac{b}{a} \biggr) \biggr]^{\alpha } \lVert u \rVert _{0}, $$

*where* $C_{\mu }$ *is the maximum value of the function*

$$ w(t, x) = \biggl(\frac{t}{x} \biggr)^{\mu } $$

*on* $[a, b] \times [a, b]$.

### Proof

Let $u \in \operatorname{AC}_{0}[a, b]$. Then

$$ u(t) = \int _{a}^{t} v(s) \,ds = \int _{a}^{t} u'(s) \,ds , \quad v(s) = u'(s) \in L[a, b], $$

and

$$\begin{aligned} \mathcal{J}^{\alpha }_{ a + , \mu } u =& \mathcal{J}^{\alpha }_{ a + , \mu } \int _{a}^{t} v(s) \,ds = \frac{1}{\Gamma (\alpha )} \int _{a}^{x} \biggl(\frac{t}{x} \biggr)^{\mu } \biggl(\log \frac{x}{t} \biggr)^{ \alpha - 1} \int _{a}^{t} v(s) \,ds \frac{d t}{t} \\ =& \frac{1}{\Gamma (\alpha )} \int _{a}^{x} v(s) \,ds \int _{s}^{x} \biggl(\frac{t}{x} \biggr)^{\mu } \biggl(\log \frac{x}{t} \biggr)^{ \alpha - 1} \frac{d t}{t}, \end{aligned}$$

by changing the order of integration. Using

$$ 0 \leq \biggl(\frac{t}{x} \biggr)^{\mu }\leq C_{\mu }, $$

we imply that

$$\begin{aligned}& \biggl\vert v(s) \int _{s}^{x} \biggl(\frac{t}{x} \biggr)^{\mu } \biggl( \log \frac{x}{t} \biggr)^{\alpha - 1} \frac{d t}{t} \biggr\vert \leq \frac{C_{\mu }}{\alpha } \bigl\vert v(s) \bigr\vert \biggl[\log \biggl(\frac{b}{a} \biggr) \biggr]^{\alpha }\in L[a, b],\quad \mbox{and} \\& \bigl\lVert \mathcal{J}^{\alpha }_{ a + , \mu } u \bigr\rVert _{0} \leq \frac{C_{\mu }}{\Gamma (\alpha + 1)} \biggl[\log \biggl( \frac{b}{a} \biggr) \biggr]^{\alpha } \lVert u \rVert _{0}. \end{aligned}$$

This completes the proof of Lemma 1.1. □

Kilbas showed the following lemma in reference [2], which is soon to be used.

### Lemma 1.2

(i) *If* $\alpha >0$, $\beta > 0$, $\mu \in R$, *and* $u \in X_{\mu }[a, b]$, *then the semigroup property holds*
  $$ \mathcal{J}^{\alpha }_{ a + , \mu } \mathcal{J}^{\beta }_{ a + , \mu } u = \mathcal{J}^{\alpha + \beta }_{ a + , \mu }u. $$
(ii) *If* $0 < \alpha < 1$ *and* $u \in \operatorname{AC}[a, b]$, *then*
  $$ \mathcal{J}^{\alpha }_{a + , \mu } \mathcal{D}^{\alpha }_{a + , \mu } u = u. $$

Let $u \in \operatorname{AC}[a, b]$ and $0 < \beta < 1$. It follows from Lemma 1.2 that

$$ \mathcal{J}^{\alpha }_{ a + , \mu } \mathcal{D}^{\beta }_{a + , \mu } u = \mathcal{J}^{\alpha - \beta }_{ a + , \mu } u $$

if $\alpha \geq \beta $.

Let $0 < \alpha _{0} < \alpha _{ 1} < \cdots < \alpha _{n} < 1$ and $0 \leq \beta _{n + 1} < \cdots < \beta _{m} \in R$, where $n = 0, 1, \ldots $ and $m > n$. In this paper, we show the uniqueness of solutions for the following new nonlinear Hadamard-type integro-differential equation for all $\mu \in R$ in the space $\operatorname{AC}_{0}[a, b]$:

1 $$\begin{aligned}& \mathcal{D}^{\alpha _{n}}_{a + , \mu } u + a_{n - 1} \mathcal{D}^{ \alpha _{n - 1}}_{a + , \mu } u + \cdots + a_{0} \mathcal{D}^{ \alpha _{0}}_{a + , \mu } u + b_{n + 1} \mathcal{J}^{\beta _{n + 1}}_{a + , \mu }u + \cdots + b_{m} \mathcal{J}^{\beta _{m}}_{a + , \mu }u \\& \quad = \int _{a}^{x} f\bigl(\tau , u'( \tau )\bigr) \,d\tau \end{aligned}$$

by Banach’s contraction principle and Babenko’s approach [4], with two applicable examples presented to illustrate the main results. It seems impossible to obtain these by any existing integral transforms or analytic local model methods. Babenko’s approach treats integral operators like variables in solving differential and integral equations. The method itself is close to the Laplace transform method in the ordinary sense, but it can be used in more cases [5, 6], such as dealing with integral or fractional differential equations with distributions whose Laplace transforms do not exist in the classical sense. Furthermore, it works well on certain differential or integral equations whose solutions cannot be achieved by the local model. Clearly, it is always necessary to show convergence of the series obtained as solutions. Recently, Li studied the generalized Abel’s integral equations of the first [7] and second kind with variable coefficients by Babenko’s technique [8–10].

It is well known that fractional calculus [3, 11, 12] has been an emergent tool which uses fractional differential and integral equations to develop more sophisticated mathematical models that can accurately describe complex systems. There are many definitions of fractional derivatives available in the literature, such as the Riemann–Liouville derivative which played an important role in the development of the theory of fractional analysis. However, the commonly used is the Hadamard fractional derivative (with $\mu = 0$) given by Hadamard in [13]. Butzer et al. [14–16] studied various properties of the Hadamard-type derivative which is more generalized than the Hadamard fractional derivative. In particular, Hadamard fractional differential equations with boundary value problems or initial conditions have been investigated by researchers using fixed point theories [17, 18]. In 2014, Thiramanus et al. [19] studied the existence and uniqueness of solutions for a fractional boundary value problem involving Hadamard differential equations of order $q \in (1, 2]$ and nonlocal fractional integral boundary conditions by fixed point theories. In 2018, Matar [20] obtained the solution of the linear equations with the initial conditions (three terms on the left-hand side at most and a given function on the right) by the parameter technique, and then investigated the existence problems of the corresponding nonlinear types of Hadamard equations using fixed point theorems. Very recently, Ding et al. [21] applied the fixed point index and nonnegative matrices to study the existence of positive solutions for a system of Hadamard-type fractional differential equations with semipositone nonlinearities. In 1967, Caputo [22] introduced another type of fractional derivative which has an advantage over R-L derivative in differential equations since it does not require to define fractional order initial conditions. Jarad et al. [23] defined the Caputo-type modification of the Hadamard fractional derivatives which preserve physically interpretable initial conditions similar to the ones in Caputo fractional derivatives. Gambo et al. [24] further presented the generalization of the fundamental theorem of fractional calculus (FTFC) in the Caputo–Hadamard setting with several new results. Adjabi et al. [25] studied Cauchy problems for a differential equation with a left Caputo–Hadamard fractional derivative in spaces of continuously differentiable functions.

There are new studies on fixed point theorems for different operators on metric spaces [26–28], as well as their applications in differential and integral equations, existence and uniqueness of solutions for equations [29–31]. Palve et al. [32] recently constructed the existence and uniqueness of solutions for the fractional implicit differential equation with boundary condition of the form

$$\begin{aligned}& {{}_{H}}D^{\alpha , \beta }_{1+} u(x) = f\bigl(x, u(x), {{}_{H}}D^{\alpha , \beta }_{1+} u(x) \bigr),\quad 0 < \alpha < 1, 0 \leq \beta \leq 1, x \in [1, b], \\& \mathcal{J}^{1 - \gamma }_{1+, 0} c_{1} u(x) + c_{2} u \bigl(b^{-}\bigr) = c_{3},\quad \alpha \leq \gamma = \alpha + \beta (1 - \alpha ), \end{aligned}$$

where ${{}_{H}}D^{\alpha , \beta }_{1+}$ is the Hilfer–Hadamard type fractional derivative of order *α* and type *β* given by

$$ {{}_{H}}D^{\alpha , \beta }_{1+} = \mathcal{J}^{\beta (n - \alpha )}_{1+, 0} D^{n} \mathcal{J}^{(1 - \beta )(n - \alpha )}_{1+, 0}, \quad n - 1 < \alpha < n, $$

and $c_{1}, c_{2}, c_{3} \in R$ with $c_{1} + c_{2} \neq 0$ and $c_{2} \neq 0$. Li [33] obtained uniqueness of solutions for the coupled system of integral equations

$$ \textstyle\begin{cases} a_{n} (\mathcal{J}^{\alpha _{n}}_{ a + , \mu } u)(x) + \cdots + a_{1} (\mathcal{J}^{\alpha _{1}}_{ a + , \mu } u)(x) + u(x) = g_{1}(x, u(x), v(x)), \\ b_{n} (\mathcal{J}^{\beta _{n}}_{ a + , \mu } v)(x) + \cdots + b_{1} (\mathcal{J}^{\beta _{1}}_{ a + , \mu } v)(x) + v(x) = g_{2}(x, u(x), v(x)), \end{cases} $$

on the product space $X_{\mu }[a, b] \times X_{\mu }[a, b]$ (it is a Banach space), based on Babenko’s approach and Banach’s contraction principle.

## Main results

### Theorem 2.1

*Assume that* $a_{i}$ *and* $b_{j}$ *for* $i = 0, 1, \ldots , n -1$ *and* $j = n +1, \ldots , m$ *are arbitrary complex numbers*, *and* $g \in \operatorname{AC}_{0}[a, b]$. *In addition*, *we let* $0 < \alpha _{0} < \alpha _{ 1} < \cdots < \alpha _{n} < 1$ *and* $0 \leq \beta _{n + 1} < \cdots < \beta _{m} \in R$, *where* $n = 0, 1, \ldots $ . *Then equation*

2 $$ \mathcal{D}^{\alpha _{n}}_{a + , \mu } u + a_{n - 1} \mathcal{D}^{ \alpha _{n - 1}}_{a + , \mu } u + \cdots + a_{0} \mathcal{D}^{ \alpha _{0}}_{a + , \mu } u + b_{n + 1} \mathcal{J}^{\beta _{n + 1}}_{a + , \mu }u + \cdots + b_{m} \mathcal{J}^{\beta _{m}}_{a + , \mu }u = g(x), $$

*has a unique solution*

3 $$ u(x) = \sum_{k = 0}^{\infty }(-1)^{k} \sum_{k_{1} + \cdots + k_{m} = k} \binom{k}{k_{1}, k_{2}, \ldots , k_{m}} a_{n - 1}^{k_{1}} \cdots b_{m}^{k_{m}} \mathcal{J}^{k_{1} (\alpha _{n } - \alpha _{n - 1}) + \cdots + k_{m}( \alpha _{n } + \beta _{m}) + \alpha _{n } }_{a + , \mu } g $$

*in the space* $\operatorname{AC}_{0}[a, b]$.

### Proof

Applying the operator $\mathcal{J}^{\alpha _{n }}_{a + , \mu }$ to both sides of equation (2), we get

$$\begin{aligned}& \mathcal{J}^{\alpha _{n }}_{a + , \mu } \mathcal{D}^{\alpha _{n}}_{a + , \mu } u + a_{n - 1} \mathcal{J}^{\alpha _{n }}_{a + , \mu } \mathcal{D}^{\alpha _{n - 1}}_{a + , \mu } u + \cdots + a_{0} \mathcal{J}^{\alpha _{n }}_{a + , \mu } \mathcal{D}^{\alpha _{0}}_{a + , \mu } u \\& \quad {} + b_{n + 1} \mathcal{J}^{\alpha _{n }}_{a + , \mu } \mathcal{J}^{ \beta _{n + 1}}_{a + , \mu }u + \cdots + b_{m} \mathcal{J}^{ \alpha _{n }}_{a + , \mu } \mathcal{J}^{\beta _{m}}_{a + , \mu }u = \mathcal{J}^{\alpha _{n }}_{a + , \mu } g. \end{aligned}$$

Using Lemma 1.2,

$$\begin{aligned}& u + a_{n - 1} \mathcal{J}^{\alpha _{n } - \alpha _{n - 1}}_{a + , \mu } u + \cdots + a_{0} \mathcal{J}^{\alpha _{n } - \alpha _{0}}_{a + , \mu } u \\& \quad {}+ b_{n + 1} \mathcal{J}^{\alpha _{n } + \beta _{n + 1}}_{a + , \mu } u + \cdots + b_{m} \mathcal{J}^{\alpha _{n } + \beta _{m}}_{a + , \mu } u = \mathcal{J}^{\alpha _{n }}_{a + , \mu } g \end{aligned}$$

by noting that $0 < \alpha _{0} < \alpha _{ 1} < \cdots < \alpha _{n} < 1$. Hence,

$$\begin{aligned}& \bigl(1 + a_{n - 1} \mathcal{J}^{\alpha _{n } - \alpha _{n - 1}}_{a + , \mu } + \cdots + a_{0} \mathcal{J}^{\alpha _{n } - \alpha _{0}}_{a + , \mu } + b_{n + 1} \mathcal{J}^{\alpha _{n } + \beta _{n + 1}}_{a + , \mu } + \cdots + b_{m} \mathcal{J}^{\alpha _{n } + \beta _{m}}_{a + , \mu } \bigr) u \\& \quad = \mathcal{J}^{\alpha _{n }}_{a + , \mu } g. \end{aligned}$$

By Babenko’s method we come to

$$\begin{aligned} u(x) = & \bigl(1 + a_{n - 1} \mathcal{J}^{\alpha _{n } - \alpha _{n - 1}}_{a + , \mu } + \cdots + b_{m} \mathcal{J}^{\alpha _{n } + \beta _{m}}_{a + , \mu } \bigr)^{-1} \mathcal{J}^{\alpha _{n }}_{a + , \mu } g \\ = & \sum_{k = 0}^{\infty }(-1)^{k} \bigl(a_{n - 1} \mathcal{J}^{ \alpha _{n } - \alpha _{n - 1}}_{a + , \mu } + \cdots + b_{m} \mathcal{J}^{\alpha _{n } + \beta _{m}}_{a + , \mu } \bigr)^{k} \mathcal{J}^{\alpha _{n }}_{a + , \mu } g \\ = & \sum_{k = 0}^{\infty }(-1)^{k} \sum_{k_{1} + \cdots + k_{m} = k} \binom{k}{k_{1}, k_{2}, \ldots , k_{m}} \bigl(a_{n - 1} \mathcal{J}^{\alpha _{n } - \alpha _{n - 1}}_{a + , \mu } \bigr)^{k_{1}} \cdots \bigl( b_{m} \mathcal{J}^{\alpha _{n } + \beta _{m}}_{a + , \mu } \bigr)^{k_{m}} \mathcal{J}^{\alpha _{n }}_{a + , \mu } g \\ = & \sum_{k = 0}^{\infty }(-1)^{k} \sum_{k_{1} + \cdots + k_{m} = k} \binom{k}{k_{1}, k_{2}, \ldots , k_{m}} a_{n - 1}^{k_{1}} \mathcal{J}^{k_{1} (\alpha _{n } - \alpha _{n - 1})}_{a + , \mu } \cdots b_{m}^{k_{m}} \mathcal{J}^{k_{m}(\alpha _{n } + \beta _{m})}_{a + , \mu } \mathcal{J}^{\alpha _{n }}_{a + , \mu } g \\ = & \sum_{k = 0}^{\infty }(-1)^{k} \sum_{k_{1} + \cdots + k_{m} = k} \binom{k}{k_{1}, k_{2}, \ldots , k_{m}} a_{n - 1}^{k_{1}} \cdots b_{m}^{k_{m}} \mathcal{J}^{k_{1} (\alpha _{n } - \alpha _{n - 1}) + \cdots + k_{m}( \alpha _{n } + \beta _{m}) + \alpha _{n } }_{a + , \mu } g, \end{aligned}$$

using Lemma 1.2 and the multinomial theorem. Clearly, $u(a) = 0$ since $\alpha _{n } > 0$ and

$$ \bigl(\mathcal{J}^{k_{1} (\alpha _{n } - \alpha _{n - 1}) + \cdots + k_{m}( \alpha _{n } + \beta _{m}) + \alpha _{n } }_{a + , \mu } g \bigr) (a) = 0. $$

It remains to show that the series converges in the space $\operatorname{AC}_{0}[a, b]$ and is absolutely continuous on $[a, b]$. By Lemma 1.1,

$$ \bigl\lVert \mathcal{J}^{k_{1} (\alpha _{n } - \alpha _{n - 1}) + \cdots + k_{m}(\alpha _{n } + \beta _{m}) + \alpha _{n }}_{ a + , \mu } g \bigr\rVert _{0} \leq K \lVert g \rVert _{0}, $$

where

$$\begin{aligned} K = & \frac{C_{\mu }}{\Gamma (k_{1} (\alpha _{n } - \alpha _{n - 1}) + \cdots + k_{m}(\alpha _{n } + \beta _{m}) + \alpha _{n } + 1)} \\ &{}\cdot \biggl(\log \frac{b}{a} \biggr)^{k_{1} (\alpha _{n } - \alpha _{n - 1}) + \cdots + k_{m}(\alpha _{n } + \beta _{m}) + \alpha _{n }}. \end{aligned}$$

Therefore,

$$\begin{aligned} \lVert u \rVert _{0} \leq &C_{\mu }\sum _{k = 0}^{\infty }\sum _{k_{1} + \cdots + k_{m} = k} \binom{k}{k_{1}, k_{2}, \ldots , k_{m}} \\ &{} \cdot \frac{ ( \vert a_{n - 1} \vert (\log \frac{b}{a} )^{\alpha _{n} - \alpha _{n - 1}} )^{k_{1}} \cdots ( \vert b_{m} \vert (\log \frac{b}{a} )^{\alpha _{n} + \beta _{m}} )^{k_{m}} }{\Gamma ( k_{1} (\alpha _{n } - \alpha _{n - 1}) + \cdots + k_{m}(\alpha _{n } + \beta _{m}) + \alpha _{n } + 1)} \lVert g \rVert _{0} \\ =& C_{\mu }E_{(\alpha _{n} - \alpha _{n - 1}, \ldots , \alpha _{n} + \beta _{m}, \alpha _{n} + 1)} \biggl( \vert a_{n - 1} \vert \biggl(\log \frac{b}{a} \biggr)^{\alpha _{n} - \alpha _{n - 1}}, \ldots , \vert b_{m} \vert \biggl(\log \frac{b}{a} \biggr)^{\alpha _{n} + \beta _{m}} \biggr) \lVert g \rVert _{0}, \end{aligned}$$

where

$$ E_{(\alpha _{n} - \alpha _{n - 1}, \ldots , \alpha _{n} + \beta _{m}, \alpha _{n} + 1)} \biggl( \vert a_{n - 1} \vert \biggl(\log \frac{b}{a} \biggr)^{ \alpha _{n} - \alpha _{n - 1}}, \ldots , \vert b_{m} \vert \biggl(\log \frac{b}{a} \biggr)^{\alpha _{n} + \beta _{m}} \biggr) < \infty $$

is the value at

$$ z_{1} = \vert a_{n - 1} \vert \biggl(\log \frac{b}{a} \biggr)^{\alpha _{n} - \alpha _{n - 1}},\qquad \ldots,\qquad z_{m} = \vert b_{m} \vert \biggl(\log \frac{b}{a} \biggr)^{\alpha _{n} + \beta _{m}} $$

of the multivariate Mittag-Leffler function $E_{(\alpha _{n} - \alpha _{n - 1}, \ldots , \alpha _{n} + \beta _{m}, \alpha _{n} + 1)}(z_{1}, \ldots , z_{m})$ given in [12]. Thus, the series on the right-hand side of equation (3) is convergent. To see $u(x)$ is absolutely continuous,

$$\begin{aligned} u(x) = & \sum_{k = 0}^{\infty }(-1)^{k} \sum_{k_{1} + \cdots + k_{m} = k} \binom{k}{k_{1}, k_{2}, \ldots , k_{m}} a_{n - 1}^{k_{1}} \cdots b_{m}^{k_{m}} \\ &{}\cdot \mathcal{J}^{k_{1} (\alpha _{n } - \alpha _{n - 1}) + \cdots + k_{m}( \alpha _{n } + \beta _{m}) + \alpha _{n } }_{a + , \mu } \int _{a}^{t} g'(s) \,ds \\ = & \sum_{k = 0}^{\infty }(-1)^{k} \sum_{k_{1} + \cdots + k_{m} = k} \binom{k}{k_{1}, k_{2}, \ldots , k_{m}} a_{n - 1}^{k_{1}} \cdots b_{m}^{k_{m}} \\ &{}\cdot \frac{1}{\Gamma (k_{1} (\alpha _{n } - \alpha _{n - 1}) + \cdots + k_{m}(\alpha _{n } + \beta _{m}) + \alpha _{n })} \int _{a}^{x} g'(s) \,ds \\ &{}\cdot \int _{s}^{x} \biggl(\frac{t}{x} \biggr)^{\mu } \biggl(\log \frac{x}{t} \biggr)^{k_{1} (\alpha _{n } - \alpha _{n - 1}) + \cdots + k_{m}(\alpha _{n } + \beta _{m}) + \alpha _{n } - 1} \frac{d t}{t} \\ = & \int _{a}^{x} \sum _{k = 0}^{\infty }(-1)^{k} \sum _{k_{1} + \cdots + k_{m} = k} \binom{k}{k_{1}, k_{2}, \ldots , k_{m}} a_{n - 1}^{k_{1}} \cdots b_{m}^{k_{m}} \\ & {}\cdot\frac{g'(s) }{\Gamma (k_{1} (\alpha _{n } - \alpha _{n - 1}) + \cdots + k_{m}(\alpha _{n } + \beta _{m}) + \alpha _{n })} \\ &{}\cdot \int _{s}^{x} \biggl(\frac{t}{x} \biggr)^{\mu } \biggl(\log \frac{x}{t} \biggr)^{k_{1} (\alpha _{n } - \alpha _{n - 1}) + \cdots + k_{m}(\alpha _{n } + \beta _{m}) + \alpha _{n } - 1} \frac{d t}{t} \,ds, \end{aligned}$$

as the function inside of the outer integral

$$\begin{aligned}& \sum_{k = 0}^{\infty }(-1)^{k} \sum_{k_{1} + \cdots + k_{m} = k} \binom{k}{k_{1}, k_{2}, \ldots , k_{m}} a_{n - 1}^{k_{1}} \cdots b_{m}^{k_{m}} \\& \quad {}\cdot \frac{g'(s) }{\Gamma (k_{1} (\alpha _{n } - \alpha _{n - 1}) + \cdots + k_{m}(\alpha _{n } + \beta _{m}) + \alpha _{n })} \\& \quad {}\cdot \int _{s}^{x} \biggl(\frac{t}{x} \biggr)^{\mu } \biggl(\log \frac{x}{t} \biggr)^{k_{1} (\alpha _{n } - \alpha _{n - 1}) + \cdots + k_{m}(\alpha _{n } + \beta _{m}) + \alpha _{n } - 1} \frac{d t}{t} \end{aligned}$$

uniformly converges with respect to *t* and belongs to $L[a, b]$ from Lemma 1.1 and the multivariate Mittag-Leffler function used above. Thus, $u(x)$ is absolutely continuous on $[a, b]$. To verify that the obtained series is a solution, we substitute it into the left-hand side of equation (2):

$$\begin{aligned}& \mathcal{D}^{\alpha _{n}}_{a + , \mu } \Biggl(\sum _{k = 0}^{\infty }(-1)^{k} \sum _{k_{1} + \cdots + k_{m} = k} \binom{k}{k_{1}, k_{2}, \ldots , k_{m}} a_{n - 1}^{k_{1}} \cdots b_{m}^{k_{m}} \\& \qquad {} \cdot \mathcal{J}^{k_{1} (\alpha _{n } - \alpha _{n - 1}) + \cdots + k_{m}( \alpha _{n } + \beta _{m}) + \alpha _{n } }_{a + , \mu } g\Biggr) \\& \qquad {}+ \Biggl(\sum_{k = 0}^{\infty }(-1)^{k} \sum_{k_{1} + \cdots + k_{m} = k} \binom{k}{k_{1}, k_{2}, \ldots , k_{m}} a_{n - 1}^{k_{1} + 1} \cdots b_{m}^{k_{m}} \\& \qquad {} \cdot \mathcal{J}^{(k_{1} +1) (\alpha _{n } - \alpha _{n - 1}) + \cdots + k_{m}(\alpha _{n } + \beta _{m}) }_{a + , \mu } g\Biggr) \\& \qquad {}+ \cdots + \Biggl(\sum_{k = 0}^{\infty }(-1)^{k} \sum_{k_{1} + \cdots + k_{m} = k} \binom{k}{k_{1}, k_{2}, \ldots , k_{m}} a_{n - 1}^{k_{1} } \cdots b_{m}^{k_{m} + 1} \\& \qquad {} \cdot \mathcal{J}^{k_{1} (\alpha _{n } - \alpha _{n - 1}) + \cdots + (k_{m} + 1)(\alpha _{n } + \beta _{m}) }_{a + , \mu } g\Biggr) \\& \quad = \mathcal{D}^{\alpha _{n}}_{a + , \mu } \Biggl(\mathcal{J}^{\alpha _{n}}_{a + , \mu } g + \sum_{k = 1}^{\infty }(-1)^{k} \sum_{k_{1} + \cdots + k_{m} = k} \binom{k}{k_{1}, k_{2}, \ldots , k_{m}} a_{n - 1}^{k_{1}} \cdots b_{m}^{k_{m}} \\& \qquad {} \cdot \mathcal{J}^{k_{1} (\alpha _{n } - \alpha _{n - 1}) + \cdots + k_{m}( \alpha _{n } + \beta _{m}) + \alpha _{n } }_{a + , \mu } g\Biggr) \\& \qquad {}+ \Biggl(\sum_{k = 0}^{\infty }(-1)^{k} \sum_{k_{1} + \cdots + k_{m} = k} \binom{k}{k_{1}, k_{2}, \ldots , k_{m}} a_{n - 1}^{k_{1} + 1} \cdots b_{m}^{k_{m}} \\& \qquad {} \cdot \mathcal{J}^{(k_{1} +1) (\alpha _{n } - \alpha _{n - 1}) + \cdots + k_{m}(\alpha _{n } + \beta _{m}) }_{a + , \mu } g\Biggr) \\& \qquad {}+ \cdots + \Biggl(\sum_{k = 0}^{\infty }(-1)^{k} \sum_{k_{1} + \cdots + k_{m} = k} \binom{k}{k_{1}, k_{2}, \ldots , k_{m}} a_{n - 1}^{k_{1} } \cdots b_{m}^{k_{m} + 1} \\& \qquad {} \cdot \mathcal{J}^{k_{1} (\alpha _{n } - \alpha _{n - 1}) + \cdots + (k_{m} + 1)(\alpha _{n } + \beta _{m}) }_{a + , \mu } g\Biggr) \\& \quad = g + \Biggl(\sum_{k = 1}^{\infty }(-1)^{k} \sum_{k_{1} + \cdots + k_{m} = k} \binom{k}{k_{1}, k_{2}, \ldots , k_{m}} a_{n - 1}^{k_{1}} \cdots b_{m}^{k_{m}} \\& \qquad {} \cdot \mathcal{J}^{k_{1} (\alpha _{n } - \alpha _{n - 1}) + \cdots + k_{m}( \alpha _{n } + \beta _{m}) }_{a + , \mu } g\Biggr) \\& \qquad {}+ \Biggl(\sum_{k = 0}^{\infty }(-1)^{k} \sum_{k_{1} + \cdots + k_{m} = k} \binom{k}{k_{1}, k_{2}, \ldots , k_{m}} a_{n - 1}^{k_{1} + 1} \cdots b_{m}^{k_{m}} \\& \qquad {} \cdot \mathcal{J}^{(k_{1} +1) (\alpha _{n } - \alpha _{n - 1}) + \cdots + k_{m}(\alpha _{n } + \beta _{m}) }_{a + , \mu } g\Biggr) \\& \qquad {}+ \cdots + \Biggl(\sum_{k = 0}^{\infty }(-1)^{k} \sum_{k_{1} + \cdots + k_{m} = k} \binom{k}{k_{1}, k_{2}, \ldots , k_{m}} a_{n - 1}^{k_{1} } \cdots b_{m}^{k_{m} + 1} \\& \qquad {} \cdot \mathcal{J}^{k_{1} (\alpha _{n } - \alpha _{n - 1}) + \cdots + (k_{m} + 1)(\alpha _{n } + \beta _{m}) }_{a + , \mu } g\Biggr) = g \end{aligned}$$

by the cancelation. Note that all series are absolutely convergent and the term rearrangements are feasible for the cancelation.

Indeed,

$$\begin{aligned}& -\sum_{k_{1} + \cdots + k_{m} = 1} \binom{k}{k_{1}, k_{2}, \ldots , k_{m}} a_{n - 1}^{k_{1}} \cdots b_{m}^{k_{m}} \mathcal{J}^{k_{1} (\alpha _{n } - \alpha _{n - 1}) + \cdots + k_{m}( \alpha _{n } + \beta _{m}) }_{a + , \mu } g \\& \qquad {}+ \sum_{k_{1} + \cdots + k_{m} = 0} \binom{k}{k_{1}, k_{2}, \ldots , k_{m}} a_{n - 1}^{k_{1} + 1} \cdots b_{m}^{k_{m}} \mathcal{J}^{(k_{1} +1) (\alpha _{n } - \alpha _{n - 1}) + \cdots + k_{m}( \alpha _{n } + \beta _{m}) }_{a + , \mu } g \\& \qquad {}+ \cdots + \sum_{k_{1} + \cdots + k_{m} = 0} \binom{k}{k_{1}, k_{2}, \ldots , k_{m}} a_{n - 1}^{k_{1} } \cdots b_{m}^{k_{m} + 1} \mathcal{J}^{k_{1} (\alpha _{n } - \alpha _{n - 1}) + \cdots + (k_{m} + 1)(\alpha _{n } + \beta _{m}) }_{a + , \mu } g \\& \quad = 0. \end{aligned}$$

The rest terms cancel each other similarly.

Clearly, the uniqueness follows immediately from the fact that the integro-differential equation

$$ \mathcal{D}^{\alpha _{n}}_{a + , \mu } u + a_{n - 1} \mathcal{D}^{ \alpha _{n - 1}}_{a + , \mu } u + \cdots + a_{0} \mathcal{D}^{ \alpha _{0}}_{a + , \mu } u + b_{n + 1} \mathcal{J}^{\beta _{n + 1}}_{a + , \mu }u + \cdots + b_{m} \mathcal{J}^{\beta _{m}}_{a + , \mu }u = 0 $$

only has solution zero by Babenko’s method. This completes the proof of Theorem 2.1. □

### Remark 1

(i) It follows from Theorem 5.3 in [2] that for $0 < \alpha < 1$
  $$ \bigl(\mathcal{D}^{\alpha }_{a + , \mu }u\bigr) (x) = \frac{x^{- \mu }}{\Gamma (1 - \alpha )} \biggl[u_{0}(a) \biggl(\log \frac{x}{a} \biggr)^{- \alpha } + \int _{a}^{x} \biggl(\log \frac{x}{t} \biggr)^{- \alpha } u_{0}'(t) \,dt \biggr], $$
  where $u_{0}(x) = x^{\mu }u(x) \in \operatorname{AC}[a, b]$. Hence, for $u \in \operatorname{AC}_{0}[a, b]$,
  $$\begin{aligned}& \bigl(\mathcal{D}^{\alpha }_{a + , \mu }u\bigr) (x) = \frac{x^{- \mu }}{\Gamma (1 - \alpha )} \int _{a}^{x} \biggl(\log \frac{x}{t} \biggr)^{- \alpha } u_{0}'(t) \,dt,\quad \mbox{and} \\& \bigl(\mathcal{D}^{\alpha }_{a + , \mu }u\bigr) (a) = 0. \end{aligned}$$
(ii) A solution of equation (2) in the space $\operatorname{AC}_{0}[a, b]$ is said to be stable if $\forall \epsilon > 0$ $\exists \delta > 0$, such that $\lVert u \rVert _{0} < \epsilon $ if $\lVert g \rVert _{0} < \delta $. Using the inequality
  4 $$\begin{aligned} \lVert u \rVert _{0} \leq & C_{\mu }E_{(\alpha _{n} - \alpha _{n - 1}, \ldots , \alpha _{n} + \beta _{m}, \alpha _{n} + 1)} \\ &{}\cdot \biggl( \vert a_{n - 1} \vert \biggl(\log \frac{b}{a} \biggr)^{\alpha _{n} - \alpha _{n - 1}}, \ldots , \vert b_{m} \vert \biggl(\log \frac{b}{a} \biggr)^{ \alpha _{n} + \beta _{m}} \biggr) \lVert g \rVert _{0}, \end{aligned}$$
  we imply that the solution *u* is stable.
(iii) The multivariate Mittag-Leffler function was initially introduced by Hadid and Luchko [34], who used it for solving linear fractional differential equations with constant coefficients by the operational method. Suthar et al. [35] studied some properties of generalized multivariate Mittag-Leffler function and established two theorems giving the image of this function under certain integral operators. Haubold et al. [36] presented a good survey of the Mittag-Leffler function, generalized Mittag-Leffler functions, Mittag-Leffler type functions, their interesting and useful properties, and applications in certain areas of physical and applied sciences. The Mittag-Leffler function plays an important role in the investigations of the fractional generalization of the kinetic equation, random walks, Lévy flights, superdiffusive transport and in the study of complex models.

Let $\nu > 0$ and $x \geq 0$. The incomplete gamma function is defined by

$$ \gamma (\nu , x) = \int _{0}^{x} t^{\nu - 1} e^{-t} \,dt. $$

From the recurrence relation [37]

$$ \gamma ( \nu + 1, x) = \nu \gamma (\nu , x) - x^{\nu }e ^{-x}, $$

we get

5 $$ \gamma (\nu , x) = x^{\nu }\Gamma (\nu ) e^{-x} \sum_{j = 0}^{\infty } \frac{x^{j}}{\Gamma (\nu + j + 1)}. $$

### Example 1

Let $0< a < x < b$. Then the Hadamard-type integro-differential equation

$$ \bigl(\mathcal{D}^{0.8}_{a + , -1}u\bigr) (x) + \bigl( \mathcal{D}^{0.7}_{a + , -1}u\bigr) (x) + \bigl( \mathcal{D}^{0.1}_{a + , -1}u\bigr) (x) + 2 \bigl( \mathcal{J}^{0.2}_{ a + , -1} u\bigr) (x) - ( \mathcal{J}_{ a + , -1} u) (x) = x^{2}, $$

has the solution

$$\begin{aligned} u(x) = & a x \sum_{k = 0}^{\infty }(-1)^{k} \sum_{k_{1} + k_{2} + k_{3} + k_{4} = k}\binom{k}{k_{1}, k_{2}, k_{3}, k_{4}} 2^{k_{3}} (-1)^{k_{4}} \\ &{}\cdot \sum_{j = 0}^{\infty } \frac{ (\log x/a )^{j + 0.1 k_{1} + 0.7 k_{2} + k_{3} + 1.8 k_{4} + 0.8} }{\Gamma (0.1 k_{1} + 0.7 k_{2} + k_{3} + 1.8 k_{4} + 0.8 + j + 1)} \end{aligned}$$

in the space $\operatorname{AC}_{0}[a, b]$. Indeed, it follows from Lemma 2.4 in [2] that

$$ \bigl(\mathcal{J}^{\alpha }_{ a + , \mu } t^{w}\bigr) (x) = \frac{\gamma (\alpha , (\mu + w) \log (x/a))}{\Gamma (\alpha )} (\mu + w)^{-\alpha } x^{w}, $$

where $\mu + w > 0$.

By Theorem 2.1,

$$\begin{aligned} u(x) = & \sum_{k = 0}^{\infty }(-1)^{k} \sum_{k_{1} + k_{2} + k_{3} + k_{4} = k}\binom{k}{k_{1}, k_{2}, k_{3}, k_{4}} 2^{k_{3}} (-1)^{k_{4}} \\ &{}\cdot \bigl(\mathcal{J}^{0.1 k_{1} + 0.7 k_{2} + k_{3} + 1.8 k_{4} + 0.8}_{ a + , - 1} t^{2}\bigr) (x) \\ = & \sum_{k = 0}^{\infty }(-1)^{k} \sum_{k_{1} + k_{2} + k_{3} + k_{4} = k}\binom{k}{k_{1}, k_{2}, k_{3}, k_{4}} 2^{k_{3}} (-1)^{k_{4}} \\ &{}\cdot \frac{\gamma (0.1 k_{1} + 0.7 k_{2} + k_{3} + 1.8 k_{4} + 0.8, \log (x/a) )}{\Gamma (0.1 k_{1} + 0.7 k_{2} + k_{3} + 1.8 k_{4} + 0.8)} x^{2}. \end{aligned}$$

Applying equation (5),

$$\begin{aligned}& \gamma \bigl(0.1 k_{1} + 0.7 k_{2} + k_{3} + 1.8 k_{4} + 0.8, \log (x/a) \bigr) \\& \quad = (\log x/a )^{0.1 k_{1} + 0.7 k_{2} + k_{3} + 1.8 k_{4} + 0.8} \Gamma (0.1 k_{1} + 0.7 k_{2} + k_{3} + 1.8 k_{4} + 0.8) \\& \qquad {}\cdot \frac{a}{x} \sum_{j = 0}^{\infty } \frac{ (\log x/a )^{j}}{\Gamma (0.1 k_{1} + 0.7 k_{2} + k_{3} + 1.8 k_{4} + 0.8 + j + 1)}. \end{aligned}$$

Thus,

$$\begin{aligned} u(x) = & a x \sum_{k = 0}^{\infty }(-1)^{k} \sum_{k_{1} + k_{2} + k_{3} + k_{4} = k}\binom{k}{k_{1}, k_{2}, k_{3}, k_{4}} 2^{k_{3}} (-1)^{k_{4}} \\ &{}\cdot \sum_{j = 0}^{\infty } \frac{ (\log x/a )^{j + 0.1 k_{1} + 0.7 k_{2} + k_{3} + 1.8 k_{4} + 0.8} }{\Gamma (0.1 k_{1} + 0.7 k_{2} + k_{3} + 1.8 k_{4} + 0.8 + j + 1)} \end{aligned}$$

is the solution in the space $\operatorname{AC}_{0}[a, b]$.

The following theorem shows the uniqueness of equation (1).

### Theorem 2.2

*Assume that* $f: [a, b] \times R \rightarrow R$ *is a continuous function*, *and there exists a constant* *C* *such that*

$$ \bigl\vert f(x, y_{1}) - f(x, y_{2}) \bigr\vert \leq C \vert y_{1} - y_{2} \vert $$

*for all* $x \in [a, b]$ *and* $y_{1}, y_{2} \in R$. *Furthermore*,

$$ C_{\mu }C E_{(\alpha _{n} - \alpha _{n - 1}, \ldots , \alpha _{n} + \beta _{m}, \alpha _{n} + 1)} \biggl( \vert a_{n - 1} \vert \biggl(\log \frac{b}{a} \biggr)^{\alpha _{n} - \alpha _{n - 1}}, \ldots , \vert b_{m} \vert \biggl(\log \frac{b}{a} \biggr)^{\alpha _{n} + \beta _{m}} \biggr) < 1. $$

*Then equation* (1) *has a unique solution in the space* $\operatorname{AC}_{0}[a, b]$ *for every* $\mu \in R$.

### Proof

Let $u \in \operatorname{AC}_{0}[a, b]$. Then

$$ \int _{a}^{x} f\bigl(\tau , u'( \tau )\bigr) \,d\tau \in \operatorname{AC}_{0}[a, b], $$

as $u'(\tau ) \in L[a, b]$ and $f(\tau , u'(\tau )) \in L[a, b]$. Clearly,

$$\begin{aligned} \biggl\lVert \int _{a}^{x} f\bigl(\tau , u'( \tau )\bigr) \,d\tau \biggr\rVert _{0} =& \int _{a}^{b} \bigl\vert f\bigl(x, u'(x)\bigr) \bigr\vert \,dx \\ \leq& \int _{a}^{b} \bigl\vert f\bigl(x, u'(x)\bigr) - f(x, 0) \bigr\vert \,dx + \int _{a}^{b} \bigl\vert f(x, 0) \bigr\vert \,dx \\ \leq& C \int _{a}^{b} \bigl\vert u'(x) \bigr\vert \,dx + \int _{a}^{b} \bigl\vert f(x, 0) \bigr\vert \,dx < \infty . \end{aligned}$$

Define a mapping *T* on $\operatorname{AC}_{0}[a, b]$ by

$$\begin{aligned} T(u) = & \sum_{k = 0}^{\infty }(-1)^{k} \sum_{k_{1} + \cdots + k_{m} = k} \binom{k}{k_{1}, k_{2}, \ldots , k_{m}} a_{n - 1}^{k_{1}} \cdots b_{m}^{k_{m}} \\ &{}\cdot \mathcal{J}^{k_{1} (\alpha _{n } - \alpha _{n - 1}) + \cdots + k_{m}( \alpha _{n } + \beta _{m}) + \alpha _{n } }_{a + , \mu } \int _{a}^{t} f \bigl( \tau , u'(\tau )\bigr) \,d\tau . \end{aligned}$$

Using inequality (4), we claim that

$$ \bigl\lVert T(u) \bigr\rVert _{0} < \infty\quad \mbox{and}\quad T(u) (a) = 0. $$

Furthermore, $T(u)$ is absolutely continuous on $[a, b]$ from the proof of Theorem 2.1. Hence, *T* is a mapping from $\operatorname{AC}_{0}[a, b]$ to $\operatorname{AC}_{0}[a, b]$. It remains to prove that *T* is contractive. Indeed,

$$\begin{aligned}& \bigl\lVert T(u) - T(v) \bigr\rVert _{0} \\& \quad \leq C_{\mu } E_{(\alpha _{n} - \alpha _{n - 1}, \ldots , \alpha _{n} + \beta _{m}, \alpha _{n} + 1)} \biggl( \vert a_{n - 1} \vert \biggl(\log \frac{b}{a} \biggr)^{\alpha _{n} - \alpha _{n - 1}}, \ldots , \vert b_{m} \vert \biggl(\log \frac{b}{a} \biggr)^{\alpha _{n} + \beta _{m}} \biggr) \\& \qquad {} \cdot \biggl\lVert \int _{a}^{t} f \bigl( \tau , u'(\tau )\bigr) \,d\tau - \int _{a}^{t} f \bigl( \tau , v'(\tau )\bigr) \,d\tau \biggr\rVert _{0}. \end{aligned}$$

Since

$$\begin{aligned} \biggl\lVert \int _{a}^{t} f \bigl( \tau , u'(\tau )\bigr) \,d\tau - \int _{a}^{t} f \bigl( \tau , v'(\tau )\bigr) \,d\tau \biggr\rVert _{0} =& \int _{a}^{b} \bigl\vert f\bigl(t, u'(t)\bigr) - f\bigl(t, v'(t)\bigr) \bigr\vert \,dt \\ \leq& C \int _{a}^{b} \bigl\vert u'(t) - v'(t) \bigr\vert \,dt = C \lVert u - v \rVert _{0}, \end{aligned}$$

we derive

$$\begin{aligned}& \bigl\lVert T(u) - T(v) \bigr\rVert _{0} \\& \quad \leq C_{\mu } C E_{(\alpha _{n} - \alpha _{n - 1}, \ldots , \alpha _{n} + \beta _{m}, \alpha _{n} + 1)} \biggl( \vert a_{n - 1} \vert \biggl(\log \frac{b}{a} \biggr)^{\alpha _{n} - \alpha _{n - 1}}, \ldots , \vert b_{m} \vert \biggl(\log \frac{b}{a} \biggr)^{\alpha _{n} + \beta _{m}} \biggr) \\& \qquad {} \cdot \lVert u - v \rVert _{0}. \end{aligned}$$

Therefore *T* is contractive. This completes the proof of Theorem 2.2. □

### Example 2

Let $a = 1$, $b = e$ and $\mu = 2$. Then there is a unique solution for the following nonlinear Hadamard-type integro-differential equation:

6 $$\begin{aligned}& \bigl(\mathcal{D}^{0.5}_{ 1 + , 2} u\bigr) (x) + \bigl(\mathcal{J}^{0.5}_{ 1 + , 2} u\bigr) (x) - \bigl(\mathcal{J}^{1.5}_{ 1 + , 2} u\bigr) (x) + \bigl( \mathcal{J}^{2.1}_{ 1 + , 2} u\bigr) (x) \\& \quad = \int _{a}^{x} \biggl(\frac{t^{2}}{C(1 + t^{100})} \sin u'(t) + \cos (\sin t) + e^{t^{2}} \biggr) \,dt, \end{aligned}$$

where the constant *C* is to be determined.

Clearly, $C_{2} = e^{2}$ is the maximum value of the function $(\frac{t}{x} )^{2}$ over the interval $[1, e] \times [1, e]$, and the function

$$ f(x , y) = \frac{x^{2}}{C(1 + x^{100})} \sin y + \cos (\sin x) + e^{x^{2}} $$

is a continuous function from $[1, e] \times R$ to *R* and satisfies

$$ \bigl\vert f(x, y_{1}) - f(x, y_{2}) \bigr\vert \leq \frac{x^{2}}{C(1 + x^{100})} \vert \sin y_{1} - \sin y_{2} \vert \leq \frac{x^{2}}{C(1 + x^{100})} \vert y_{1} - y_{2} \vert \leq \frac{1}{C} \vert y_{1} - y_{2} \vert . $$

Obviously $\log b/a = 1$. By Theorem 2.2, we need to calculate the value

$$\begin{aligned}& \sum_{k = 0}^{\infty }\sum _{k_{1} + k_{2} + k_{3} = k} \binom{k}{k_{1}, k_{2}, k_{3}} \frac{1}{\Gamma (k_{1} + 2 k_{2} + 2.6 k_{2} + 1.5)} \\& \quad = \sum_{k = 0}^{\infty }\sum _{k_{1} + k_{2} + k_{3} = k} \binom{k}{k_{1}, k_{2}, k_{3}} \frac{1}{\Gamma (k + 1.5 + k_{2} + 1.6k_{3})} \\& \quad = \frac{1}{\Gamma (1.5) } + \sum_{k = 1}^{\infty } \sum_{k_{1} + k_{2} + k_{3} = k} \binom{k}{k_{1}, k_{2}, k_{3}} \frac{1}{\Gamma (k + 1.5 + k_{2} + 1.6k_{3})}. \end{aligned}$$

For $k \geq 1$,

$$ \frac{1}{\Gamma (k + 1.5 + k_{2} + 1.6k_{3})} \leq \frac{1}{\Gamma (k + 1) } = \frac{1}{k!}, \quad \mbox{and}\quad \sum_{k_{1} + k_{2} + k_{3} = k} \binom{k}{k_{1}, k_{2}, k_{3}} = 3^{k}. $$

Therefore,

$$\begin{aligned}& \sum_{k = 0}^{\infty }\sum _{k_{1} + k_{2} + k_{3} = k} \binom{k}{k_{1}, k_{2}, k_{3}} \frac{1}{\Gamma (k_{1} + 2 k_{2} + 2.6 k_{2} + 1.5)} \leq \frac{1}{\Gamma (1.5) } + \sum_{k = 1}^{\infty } \frac{3^{k}}{k!} \\& \quad < \frac{1}{2} + \sum_{k = 0}^{\infty } \frac{3^{k}}{k!}. \end{aligned}$$

Then, choose a positive *C* such that

$$ C e^{2} \Biggl(\frac{1}{2} + \sum _{k = 0}^{\infty }\frac{3^{k}}{k!} \Biggr) < 1. $$

By Theorem 2.2, equation (6) has a unique solution. We note that the series $\sum_{k = 0}^{\infty }\frac{3^{k}}{k!} $ converges.

## Conclusions

Using Babenko’s approach and Banach’s contraction principle, we have derived the uniqueness of solutions for the new nonlinear Hadamard-type integro-differential equation for all $\mu \in R$:

$$\begin{aligned}& \mathcal{D}^{\alpha _{n}}_{a + , \mu } u + a_{n - 1} \mathcal{D}^{ \alpha _{n - 1}}_{a + , \mu } u + \cdots + a_{0} \mathcal{D}^{ \alpha _{0}}_{a + , \mu } u + b_{n + 1} \mathcal{J}^{\beta _{n + 1}}_{a + , \mu }u + \cdots + b_{m} \mathcal{J}^{\beta _{m}}_{a + , \mu }u \\& \quad = \int _{a}^{x} f\bigl(\tau , u'( \tau )\bigr) \,d\tau \end{aligned}$$

in the Banach space $\operatorname{AC}_{0}[a, b]$, with two examples given to illustrate the main theorems. The results obtained are fresh in the present studies, and they cannot be achieved via any existing integral transforms or local model methods to the best knowledge of the author.

## Data Availability

Not applicable.
